# Coping Dynamics of Consulting Psychology Doctoral Students Transitioning a Professional Role Identity: A Systems Psychodynamic Perspective

**DOI:** 10.3390/ijerph17155492

**Published:** 2020-07-30

**Authors:** Antoni Barnard, Aden-Paul Flotman

**Affiliations:** Department of Industrial & Organisational Psychology, UNISA, Pretoria 0003, South Africa; flotma@unisa.ac.za

**Keywords:** consulting psychology, coping, defences, identity work, identity tension, professional identity, system psychodynamic

## Abstract

To remain relevant and valuable, the psychology profession in South Africa continues to transform and evolve in response to the changing needs of society. Some psychologists embark on development opportunities to advance their professional qualifications and skills. In doing so, they experience identity tensions inherent to professional identity development and transformation. Understanding how psychologists cope with professional identity transition will enable them to develop a self-efficacious service offering and broaden the reach of psychology in the South African context. The aim of this study was to explore the identity work of a group of eight consulting psychology doctoral students to develop a system psychodynamic understanding of their coping dynamics while transitioning to a professional role identity. Students’ self-reflective essays about becoming a consulting psychologist constituted the data protocols for the study and were analysed through hermeneutic phenomenological analysis. Findings describe how students cope with performance and survival anxieties through anti-task behaviour and immature as well as sophisticated psychodynamic defences. The study contributes to the exploration of the coping concept and its manifestation, by proposing defensive coping as a natural dynamic phenomenon in the process of adapting to a transforming professional identity.

## 1. Introduction

Twenty-five years post-apartheid the profession of psychology in South Africa has been beset by identity tensions regarding its relevance and value to a continuously transforming South African society [1,2,3]. The identity dilemmas of the psychology profession seem evident in the disagreement among its practitioners about changing the Regulations Defining the Scope of the Profession of Psychology. The original regulations defined a scope of practice for psychologists registered with the Health Professions Council of South Africa (HPCSA) to distinguish the different categories of counselling: industrial and organisational (IO), clinical, educational and research. The original regulations were however amended in 2011 without an adequate consultation process. Unlike the original widely accepted demarcated scope of practice, the amendments narrowed down practice opportunities for some of the psychology categories and seemed more favourable to others. The process was criticized for being forced upon the profession, for creating power disparities between the different categories of psychology and for marginalising certain practitioners, preventing them from contributing to sectors in society in dire need of mental health and well-being [1,4]. A large section of the profession was therefore dissatisfied and angry, resulting in a court order on 14 November 2016 by the High Court of South Africa (Western Cape Division, Case No: 12420/13) declaring the amended regulations invalid [5]. A notice by the Department of Health not to proceed with any amendments that were recently published [6] declared the original Regulations Defining the Scope of the Profession of Psychology of 2008 to remain in force.

Disputes about the amended scope of practice reflected underlying identity tensions within the psychology fraternity. On the positive side, the discussion also aligned with evolving frames of thought about the profession and its impact on society at large and about opportunities for continued professional development and multidisciplinary collaboration. One such opportunity was created in a doctoral programme specialising in consulting psychology. The programme is offered in collaboration by the Departments of Psychology and IO Psychology at UNISA. Students admitted to the programme include registered and practising psychologists across the five different HPCSA registration categories. These educational, clinical, IO, counselling or research psychologists entering the field of consulting psychology invariably experience a role identity transition [7] or an altering of their professional identity [8].

When transitioning to a new role, people naturally experience identity tensions between previously known meanings of the self and new role expectations [9]. They attempt to resolve these tensions by engaging in identity work [10]. Identity work entails the restructuring, reframing and development of identity meanings that constitute the self in specific social contexts [11,12,13]. It refers to the intrapersonal process in which individuals engage to form, repair, maintain, strengthen or revise their identities [14]. Identity work is therefore relevant to intrapersonal coping, because it enables individuals to cope with difficult work-life demands [10,15] and adjust to important work-life transitions [16,17]. Breakwell introduced the idea that identity work is based on intrapsychic and interpersonal coping strategies [9]. Observation of identity work is therefore expected to reveal the intrapersonal coping strategies that individuals apply to resolve the identity tensions they experience consequent to role transitions.

The aim of this study was to develop an understanding of the coping dynamics that consulting psychology doctoral students manifest when transitioning their professional role identity. Understanding how the students cope with the identity tensions they experience will enable them to develop a self-efficacious service offering to their clients and broaden the reach of psychology in the South African context. Identity work is a natural and involuntary [7] or subconscious [18] process, which in the context of this study happens in both the individual student and in the bigger systemic context of the psychology fraternity. It was therefore deemed useful to explore coping with transitioning a professional identity by applying a systems psychodynamic perspective.

## 2. Literature Review

### 2.1. System Psychodynamics

Systems psychodynamics is rooted in psychoanalysis, object relations, systems theory and the Tavistock Human Institute of Human Relations [19,20]. Although Freud was the father of psychoanalysis, it was Klein’s object relations theory, group relations and open systems theory that contributed significantly to the systems psychodynamic paradigm [21,22]. The systems psychodynamic approach developed as a suitable stance to explore beneath-the-surface behaviour in organisations [23] and to the study of organisational change dynamics [24,25]. The central tenets of system psychodynamics lie in the semantic co-occurrence of the words systems and psychodynamic. Open systems principles are firstly applied in understanding behaviour, and individual behaviour is regarded as a function of systemic dynamics as much as it is a representation of (mirroring) systemic behaviour. Secondly, psychodynamic theory represents a fundamental focus on unconscious behavioural dynamics and includes the spectrum of psychoanalytic perspectives on individual and social experiences and mental processes. This includes un understanding of anxiety as the basis of group and unconscious systemic behaviour [26,27] and the involuntary use of defence mechanisms to cope with anxiety [28].

Central to psychodynamic thinking is the assumption that part of the mental life of individuals is hidden and affects them in ways of which they are not always aware—the unconscious life [29]. Furthermore, in system psychodynamics, anxiety is regarded as the driving force of all relational dynamics in a system, as any change is seen to arouse anxiety naturally [25]. Anxiety manifests itself as personal anxiety, task-related anxiety or role anxiety but always in a systems context and representative of larger anxieties in the system [26]. Typically, transitions in the work environment arouse performance and survival anxiety [30]. In response to this anxiety, defence mechanisms are used unconsciously [20,31] to remain in control and to experience a sense of safety, security and acceptance [27,32].

Defences are fundamental to coping in psychodynamic theory [33,34,35]. In systems psychodynamics, defences manifest themselves as basic assumption behaviour, which includes dependence, fight-flight, pairing [21], one-ness, we-ness and me-ness [20]; in primary defences such as splitting, introjection, suppression, denial, projection and projective identification [22,28,36] and in more sophisticated defences such as rationalisation and intellectualisation [37]. Defences operate on a continuum, ranging from primitive, debilitating impairments to more sophisticated competence-enhancing adaptations [25,38]. These defence mechanisms have a protective function and are neither good nor bad [39]. Individuals develop defences as coping strategies from an early age to deal with reality and to maintain a functional sense of self [28].

There is a longstanding link between coping theory and psychodynamics [40], yet contemporary perspectives on coping strategies typically focus on cognitive-behavioural perspectives such as emotion-focussed, problem-focussed, social-support coping and religious coping [41]. Such coping strategies have been distinguished as involving conscious, purposeful effort [33]. This paper takes the stance that coping is a process of psychological adjustment [42,43] that includes conscious and unconscious coping as means of adaptation [33,34,44]. Applying a system psychodynamic stance to understand coping is of value, since it provides a more holistic and systemic understanding of coping behaviour [45] and uncovers the unconscious as a creative source of knowledge [23,46]. When experiences are explored from a systems psychodynamic stance, one can enhance awareness, understanding and learning of both conscious and unconscious dynamics [47,48]. Such knowledge is deemed essential in managing and facilitating real behavioural change [25] and prevents anxiety and defences from becoming destructive in the workplace [49].

### 2.2. Systems Psychodynamics and Identity Work

Systems psychodynamics approaches identity firstly from a group relations stance, relating the individual’s role identity to a systemic role identity [22]. From this perspective, taking up a new role entails a psychosocial dynamic that emerges from the interface between the person and the formal role as defined by a particular context [50]. As such, viewed from a psychodynamic perspective, identity work involves cognitive, emotional and social processes that are social or systemic in nature (i.e., cannot be done in isolation) [12,13], and such identity work is often stimulated by anxiety [51].

System psychodynamics further focus on understanding unconscious experience. In doing identity work, a person therefore consciously, but also unconsciously, negotiates personal beliefs, needs and aspirations in adjusting to latent role demands. Projective processes that relate to psychodynamic defence dynamics, in the form of projective identification, transference and counter-transference, are benign parts to be worked with by individuals in the process of identity work [52]. Psychodynamically, identity work is therefore essential in everyday coping with tensions in the self and maintaining well-being [44].

A psychodynamic lens to identity enriches our understanding of identity work beyond current identity theory [44]. System psychodynamic literature emphasises that creating reflective spaces is essential to facilitate constructive role transition [49,53]. In this regard and relevant to the consulting psychology programme context of this study, the concept of identity workspaces refers to institutions that provide a holding environment for individuals to do their identity work [54]. This holding environment serves as a social context that not only reduces distressing emotions but also actively facilitates sense-making, either aimed at identity stabilisation or identity transition [55].

A significant portion of identity work is emotional, dynamic and complex and lies beneath the surface of conscious behaviour [18,44]. Taking a systems psychodynamic stance stresses that this intrapsychic dimension of identity work must also be worked with. Thus, by “focusing on the ongoing dynamic interaction of individual and social, cognitive and emotional, conscious and unconscious factors, the system psychodynamic perspective is particularly well suited to enriching our understanding of identity and identification” [54].

## 3. Materials and Methods

This paper is part of a larger project studying the experiences of master’s and doctoral students to improve academic curricula and the throughput of postgraduate students. The next sections give an outline of the research methodology that directed the study, an explication of the research setting, the data protocols and participants, as well as the analytic strategy applied.

### 3.1. Research Methodology

A qualitative inquiry was deemed most appropriate for the exploratory and descriptive nature of the study. The study followed a hermeneutic phenomenological approach in its overall design. In hermeneutic phenomenology knowledge generation equates to the co-construction of meaning between the researcher and the researched [56]. It allows for ascribing meaning to participants’ experiences through critical interpretation, inevitably influenced by researcher experience and theoretical preconceptions [57,58,59]. Fundamentally, from a hermeneutic phenomenological perspective, researchers’ critical interpretation of participants’ phenomenological experiences is imperative for rigorous scientific research [60]. To achieve the stated objective, researchers’ interpretations were influenced in particular by applying a systems psychodynamic lens or meta-theoretical orientation during data analysis. System psychodynamics refers to an extensive body of scholarship [46] that provides a sound meta-theory to develop truthful, useful and credible insights and hypotheses [48]. Both researchers (a black man and a white woman) hold PhDs in psychology and are part of faculty in an open-distance, e-learning tertiary institution. They are registered psychologists with the HPCSA with a keen research interest in socio-analytic research methodologies, hermeneutically informed by system psychodynamics. They are also both involved in the teaching of the course work component of the Consulting Psychology doctoral programme.

### 3.2. Research Setting

The doctoral programme in Consulting Psychology offered at a tertiary open distance and e-learning (ODeL) institution in South Africa constituted the setting for this research. The institution’s two main campuses are located in the Gauteng region, with satellite campuses across South Africa. Gauteng is the smallest province yet the most densely populated (742.6 per square metre) in South Africa, accommodating 1.349 million of the 55.9 million South Africans (https://www.southafricanmi.com/sa-by-numbers.html). National and international student enrolments at this ODeL institution varied between 354,743 and 381,483 in the 2015 to 2018 registration periods. The Consulting Psychology doctoral programme has enrolled eight to 12 students per annum since its inception in 2005. The first year of the programme entails course work involving 11 focus areas, of which one focusses on consulting as process. The learning outcome of this focus area is intended to develop students’ capability to explore their consulting profile and develop a personal frame of reference for consulting. During the course work component of the Consulting Psychology doctoral programme, students attend five block weeks of face-to-face training and complete several individual and group-based projects as part of their formative assessment. Another primary focus of the first year is the successful defence of a research proposal, which forms the basis for continued research-based study from the second year until completion of the degree.

### 3.3. Data Protocols

One of the individual tasks students are required to do in their first year of study is to keep a personal diary in which they journal about becoming a consulting psychologist. The students then write self-reflective essays that entail a summative and critical reflection based on periods of five and eight months’ journaling. To explore the coping dynamics of students transitioning into a consulting psychology role, self-reflective essays constituted the data protocols for this study. Critical reflexivity refers to the process in which one questions one’s positionality and basic assumptions about life, people and oneself, while seeking alternative ways of being and looking at things [61,62,63]. In this regard, self-reflective practice is akin to identity work as defined by Sveningson and Alvesson [14]. In Nagata’s definition of self-reflexivity [64], coping as an adaptational endeavour underlying identity work is furthermore evident. He defined self-reflexivity as having a conversation with the self about one’s experiences while one is experiencing them, leading to the ability to regulate internal psychological and unconsciously driven responses. Self-reflective essays were moreover deemed appropriate to the purpose and system psychodynamic orientation of this study, as reflective practice elicits a meta-level of thinking and feeling about the self [64] and makes the “unthought known” observable and conscious [65].

Instructions for the self-reflective essays were based on circular existential and relational questions relevant to experiential learning activities aimed at developing critical reflexivity [61]. The questions were formulated as a guide to facilitate critical self-reflection in line with the objective to engage with and develop a personal frame of reference as a consulting psychologist. The intention with these questions was not to be prescriptive but to offer some direction to the task of self-reflecting. The first question aligns with Cunliffe’s existential (who am I?) and relational (who am I in relation to something/someone?) approach [61] and asks the student to reflect on the question: Where am I at this moment regarding my personal frame of reference? The second and third questions refer to the circular influence approach [61], examining how existential and relational learnings influence one’s responses and ways of acting, behaving and responding. The second question asks: How does your evolving personal frame of reference affect your understanding of the landscape of consulting psychology? The third is: What is the impact of your evolving personal frame of reference on your personal consulting profile?

Self-reflective essays are shared with lecturers after five months’ journaling and again after another three months’ journaling. Through purposive, convenience sampling, the transcripts that constituted the data sets for this study included all the self-reflective essays of the current course work students after eight months. By that time, the students had already completed most of their course work-related projects and workshops.

### 3.4. Participants

To ensure the confidentiality and anonymity of participants, they are not identified as individuals but described as a group. The participants included three women and five men. In terms of population group, three were white, one was Indian and four were black, with an age range of 29–58, and average age of 40. Participants were approached face to face during their first block period on campus, and the nature and purpose of the research were explained to them. This was followed up by individual e-mails requesting their participation. Inclusion criteria entailed that participants had to have a master’s degree in a specific psychology domain; be registered psychologists with the HPCSA, practising for at least three years; be registered doctoral students in Consulting Psychology; be willing to share their self-reflective experiences and be willing to participate in the research. Four participants were registered in the category of IO psychology, and one each was registered as clinical, educational, counselling and research psychologist. All the participants had gained a minimum of three years’ experience since obtaining their professional HPCSA registration. Pseudonyms are used in the findings to indicate the number of the participant, for example, P4 refers to participant number four.

### 3.5. Ethical Considerations

Ethics approval was obtained from the relevant Institutional Senate Ethics Committee (REF#:2017_RPCS_018). Participants consented in writing that data generated from their self-reflective work on being a consulting psychologist could be used for research purposes relevant to the bigger project. The researchers abide by the ethical codes of conduct as prescribed by the UNISA ethics policy, as well as the ethical code of psychologists registered under the HPCSA. The researchers also declare that the research was conducted in compliance with the ethical principles enunciated in the Declaration of Helsinki. As such, ethical principles of anonymity, confidentiality and informed consent were upheld by reporting on the data in a collective, interpretive sense and by using pseudonyms.

### 3.6. Data Analysis

Eight self-reflective essays were loaded as primary data documents in Atlas.ti to ease the management of the data analysis process. Data were analysed through hermeneutic phenomenological analysis according to the analytic stages of naïve reading, structural thematic analysis and comprehensive understanding [66]. The fundamental premise of the hermeneutic circle was applied throughout these stages of analysis. Initially, we considered each individual’s experience in relation to the meaning we constructed from the participants’ collective experience and vice versa [26]. We also compared findings from each stage with findings in the previous stage in a consistent, circular manner [67]. At this point the themes provided a richness in terms of saturation and no more themes emerged to add to the clear description of coping dynamics from a system psychodynamic perspective.

## 4. Findings

The naïve reading revealed how some students struggled to engage with the task of self-reflection in that they recited textbook definitions of the skills and competencies of a consulting psychologist. Without reflecting on their emotional responses to the personal development journey they were on, they merely noted that they were busy developing the mentioned list of skills and competencies. Others provided less detached accounts of their experience of becoming a consulting psychologist, but their performance anxiety in relation to the task topic of becoming a consulting psychologist was evident in how they engaged in anti-task behaviour. Performance anxiety was coped with in various ways, mostly to avoid the required identity work or to resist the professional identity role transformation they were experiencing. Especially, students who did not engage in self-reflection consistently throughout the year seemed to struggle with performance anxieties and integrating identity tensions. Three themes were constructed in the thematic analysis to describe the students’ coping with the identity tensions and demands from a systems psychodynamic perspective. The first theme described how they resisted the primary task of self-reflection and, consequently, resisted identity work through basic assumption (anti-task) behaviour. The second theme described their coping dynamics by applying primary defences against the perceived incongruence of conscious (normative) and unconscious (phenomenological and existential) roles. The third theme described their coping along the more sophisticated defences the students applied to resolve the identity tensions and performance anxiety they experienced. The three themes are conceptualized below in terms of their different sub-themes and related categories. Verbatim data from the participants’ narratives are used to illustrate the meaning-making during analysis.

### 4.1. Resisting the Primary Task through Basic Assumption Behaviour

The first sub-theme entails resisting the task of deep self-reflection and identity work through basic assumption (anti-task) behaviour, which manifested in dependency, fight behaviour, pairing and me-ness.

#### 4.1.1. Dependency

Students idealised several external objects such as lecturers, the programme and consulting knowledge. In doing so, they expressed their dependency on external structures and authority figures to feel successful in their career or profession. The Consulting Psychology doctoral programme was related to the mother figure and several instances of how the programme or lecturers or the consulting psychology knowledge domain was made the hero or saviour were evident in the data. Such “heroing” usually followed students’ expressed feelings of insecurity, low self-confidence and uncertainty or the need to succeed or fantasise about career opportunities and personal change. In this way, some students seemed to cope by relying on the doctoral programme to help them deal with the identity transition they were seeking and working with, while doing the task of self-reflection in the role of becoming a consulting psychologist. Table 1 below summarises pertinent verbatim extracts supporting how students acted out or expressed their dependency in an effort to cope with the identity tensions and insecurities they were experiencing.

#### 4.1.2. Fight

Fight reactions were a way of coping with the need to preserve the self and resist the identity tensions felt during self-reflection. One student attacked the task of self-reflection by denigrating its value in light of preserving what she feels more comfortable with, namely to be a practitioner: “When confronted by theory and philosophy, my question is always what does this look like in practice? If I can’t figure that out, or it is not clear to me, it is not useful, no matter how beautiful or elegant it is” (P1). Another student demonstrated fight behaviour by attacking team work as irreconcilable with her leadership style. Again, the student is trying to preserve her known leadership identity. She reveals the identity tension she experiences and demonstrates how she copes with it through fight behaviour: “I naturally take a leadership role (my current role at work requires this) and develop innovative ideas in a team setting; this had to be suppressed at times” (P2).

#### 4.1.3. Pairing

Students attempted to pair with an authoritative form or powerful other, by aligning themselves with the programme, with a lecturer or with the class as a team. In expressing her need to be part of the group, P2 copes with the anxiety she experiences in transitioning into the consulting psychologist role: “after the week, I realised that I needed the group. In May, I became more involved with the team, working in groups. Even though it was a bit uncomfortable for me working as a member of a team, I did not just play my role but enjoyed it. Furthermore, the course seemed easier as we shared similar experience and could relate.” Several students strongly aligned themselves to the doctoral programme and the consulting psychology knowledge domain, proclaiming a new-found sense of confidence and security in their own abilities. P4, for example, states: “this programme has broadened my horizons. It has help[ed] me to appreciate that there are three different levels of intervention that any qualified and properly trained psychologist must be able to operate in, namely, individual, group and organisational level. The above, is an illustration of the significant impact that this programme is having on me as a professional”. Similarly, P8 notes that she feels more confident as a psychologist because “there is no better programme I would recommend to my peers and colleagues than that of the Consulting PhD programme”. She continues to align herself to the group in order to cope with the potential loneliness that she experiences in the identity transition process that she embarked on: “My classmates and I have an exceptional relationship where we assist each other not only academically but also personally and professionally.” Through pairing, it seems that the students constructively cope with the performance anxieties that emanate from the identity demands they experience in the context of the consulting psychology doctoral programme.

#### 4.1.4. Me-Ness

Students experience tension between the identity needs of belonging and of uniqueness. The push and pull between wanting to retain individuality, while wanting to be part of the group, was in particular coped with by emphasising me-ness. Through me-ness, students cope with identity transition, as is seen in a student’s denial of personal change: “Looking at my framework of reference when I started the programme, I could not really say that much has changed with regards to my view My frame of reference is still focused on the individual but not excluding the group and organisational factors” (P5). Similarly, P1 notes that she also does not require much personal change and therefore does not see the need for the task of self-reflection: “Reflecting on my personal frame of reference is not something that I usually do, and I am not sure whether I will ever become in the habit of doing so. I like to think that I have a degree of self-awareness”. In this way, students resist the task of self-reflection, and in doing so, they are resisting identity work by highlighting their inner strength and adequacy. By resisting the task in this way, students reveal the anxiety they experience when engaging in a task focussed on identity work, a task that uncovers identity tensions consequent to transforming their professional identity. Me-ness illustrates the individual’s escape into the inner world that is felt to be safe, comfortable and good [68,69]. Coping with the task of self-reflection (identity work) through self-reliance, independence and distancing from interdependence is evident in the words of P2: “After working for almost fourteen years in management and being offered directorship at the age of thirty-eight years, I began to realise that I needed to trust my capabilities, to assure myself that I have what it takes to ‘manage’ whatever comes my way” and “The realisation has to some extent shaped a frame of reference for my believing that I do not necessarily need others: I cannot/do not like working as a member of a team, I do not like group work, as I find that I achieve better results, when I complete tasks in my own way and independently.”

### 4.2. Primary Defences Defending against Perceived Identity Incongruences

The second theme entails defending against perceived identity incongruences. The perception of identity incongruence was evident in students experiencing tension between their conscious (normative) and unconscious (phenomenological and existential) roles. Coping with the resulting performance anxiety was evident in primary psychodynamic defences such as splitting, projection and projective identification [25]. These have also been referred to as immature defences [70].

#### 4.2.1. Splitting

To deal with the anxiety elicited in the task of self-reflection, students tended to split objects of their role identification into good and bad opposites. One split was evident in juxtaposing the academic role with the role of practitioner, projecting onto the academic role the less worthy and valuable task:

I am not sure that the academic world will ever be home to me. It might become easier for me to understand and adhere to its customs, but I don’t think I will ever choose to stay there for longer than I need to. I would rather be in the world outside where I can be doing. I have always performed well academically, and everyone has expected that I would follow an academic career, but that did not interest me. The theoretical and philosophical have never appealed to me, the practical did, and that is where I positioned myself (P1).

Similarly, P6 splits the roles of scientist and practitioner yet in a less obvious manner. For the greater part of his essay, he has copied definitions of the scientist-practitioner roles and aligned himself to each definition, without demonstrating authentic reflection on how he engages with and integrates these roles. His stance remains distanced and detached, as seen in his paraphrased list of roles: “My consulting value proposition and paradigm as a scientist-practitioner is adapting and designing assessment technologies/instruments for purpose of selection, training and vocational assessment; conducting individual assessment for the purpose of selection and training, career and vocational guidance, and leadership coaching; conducting individual assessment on individual wellness and work adjustment (psychopathology and work adjustment); conducting career counselling, advice and therapy ” (P6).

Another student copes by creating a split between the consulting psychologist and other “external forces” that inhibit constructive work in the organisation. In doing so, P5 reveals the performance anxiety he experiences in taking up the consulting psychologist role: “I also realise that there are forces internal and external that would make it more challenging for the Consulting Psychologist to find a balance between the organisation, the group and the individual. The challenge for the Consulting Psychologist is to divorce him/herself from these external forces such as politics”. The spilt in professional roles is also evident in P4’s hero-ing of the consulting psychologist role as one that “is equipped with diverse knowledge and experience to help others meaningfully” as opposed to “I have always restricted my dealings with issues and clients strictly to methodologies within industrial psychologist stream.”

In working with the tension between her need for belonging and her need for independence, P2 copes by splitting the self from the group: “I found myself concerned with whether the members of the team were up to date with expectations and going to an extent of contacting them through emails and some with a telephonic discussion. I consider this a degree of improvement as at least I thought about the team and did something about that thought”. Splits were also evident in the different professional categories of psychology: “I have always recognised myself as a clinician who works only with the individual to target the presented pathology/illness for a desired behaviour, treatment management and curative modalities … Consulting Psychology is different” (P8).

Splitting seemed to reveal students’ perception of identity incongruence in what they normatively expected themselves to do in the role, as opposed to their inherent and obscured insecurities and fears of incompetence. Their conscious and unconscious self-expectations left them with anxious feelings, which they resolved by creating outside forces that were feared or not acceptable. The way participants cope through splitting becomes more evident when integrated with how they invariably use projection as an unconscious defensive coping strategy.

#### 4.2.2. Projection

Engaging in the self-reflective exercise created discomfort because it entailed having to face and work with both positive and negative aspects of the self. Students defended themselves against this discomfort by attributing negative parts of the self to others. Narcissism and self-indulgence were projected onto the psychology profession as a whole and onto the task of reflecting on personal paradigms: “My impression is that psychologists have become so obsessed with their own paradigms and frames of reference, and in the process became so self-focused, that they are forgetting why they are here. There is a whole country in dire need of psychological services, but psychologists are pre-occupied with their paradigms, like Nero fiddling while Rome is burning” (P1). Later this student also projects egotism onto the corporate world: “The corporate world needs to learn how to effectively engage with communities, even if it [is] just for the benefit of their own triple bottom line—let’s not kid ourselves, the corporate world rarely does something out of pure altruism” (P1).

Students cope with their performance anxiety by projecting inefficiency onto colleagues: “I often have a feeling that it will be better if I can do my work without interruptions from colleagues” (P2). They cope with their own fear of engaging in the self-reflection task (identity work) by projecting the fear of change onto organisations and “other people”: “Some organizations may initially be apprehensive of change. The apprehension is normal… they do not want change because change requires them to work and use energy, time and effort” (P8).

Positive attributes were also projected onto the consulting psychology profession and the consulting psychology doctorate. In a way, the students were idealising consulting psychology as an all-knowing, super-capable and competent force, which they wanted to attain: “although my frame of reference has been limited to models within industrial psychology, this programme has broadened my horizons” (P4). Similarly, P3 projects his ideal self onto the programme: “I feel that my journey through the PhD programme … will assist in refining my work as a scientific-practitioner.”

#### 4.2.3. Projective Identification

Students’ projective identification was manifested predominantly in identifying with the sense in the class that their careers and professional competence would be adequate if they had *enough* knowledge. The students’ projective identification started with them having introjected feelings of inadequacy, low self-efficacy and a sense of knowing too little and having too little experience. P3 comments “Starting off, my point of reference with regard to consulting psychology was nothing” and P2 reflects “I reflected on what I associated the term, capability, with. My impression is that there had been a rooted feeling of doubt, not only self-doubt, but silently kept childhood statements that echoed: ‘you will not be able to be on the same level with your class/group’.”

The introjected inadequacy led to the fantasy that the doctoral programme would empower them to become successful consulting psychologists (their expressed normative role). Students’ valence for thinking they did not know enough was further evident in expressions of rigid self-expectation: “Some workshops really started providing me with more insights in what a Consulting Psychologist is supposed to know .... Consequently, as a Consulting Psychologist I need to become more aware of how the environment, the culture and the political spectrum dictates the functioning of the organisation, secondly I need to be able to understand ...” (P5). Similarly, P8 says: “Consulting Psychology is comprehensive and quite theoretical …. Consulting psychologists need stringent and empirically tested interventions and methods to be able to target these dynamics.”

### 4.3. Applying Sophisticated Defences to Cope with Identity Tension and Performance Anxiety

In psychodynamic theory, rationalisation and intellectualisation are regarded as mature defence mechanisms [45,71]. Through these mechanisms, the students suppressed emotions and proposed rational arguments to explain or describe their experiences.

#### 4.3.1. Intellectualisation

Examples of intellectualisation, where students suppress their emotional experiences, abound. One student, P4, engages with identity work (self-reflection) by speaking about her experience of becoming a consulting psychologist in a detached (theoretical) and absolute (over-generalising) manner: “A consulting psychologist must always be aware of the values and past experiences that he or she brings into the system or relationship with the new client. I have learnt that this helps for one to remain accurate and objective on matters while dealing with the client”. Student P8 addresses her fear of incompetence by also providing a rational over-generalisation of what one *should* do when engaging in identity work: “It is important to also be aware of one’s biases, personality and behavioural dynamics regarding the process as a psychologist and not allow these to hinder the consulting process”. In a critical tone, using absolute terms, P1 defends against engaging personally with the identity transformation: “As a consultant, my work must be practical and useful, yet imbedded in science, and I must be aware of my impact and others’ impact on me. Doing this programme is a time for me to grow and develop and acquire new skills.”

Intellectualising their personal development in becoming a consulting psychologist, students avoid facing the anxiety that stems from such very personal identity work. In this way, they suppress feelings of discomfort experienced in relation to their evolving and transforming professional identity.

#### 4.3.2. Rationalisation

The belief that knowledge imparted to students by the lecturers and the programme will enable success and competence was found in various essays. This belief shows how students idealise the programme and knowledge as an intellectual defence against the fear of incompetence. In presenting an essay that is fully paraphrased from competency lists and definitions pertaining to consulting psychology, P6 rationalises his professional development and does not engage in self-reflection at all. Also finding solace in knowledge and science, P3 rationalises that the knowledge he will gain as a result of the programme will make him a more effective psychologist: “This potential is where positive psychology can be enlisted as a new frame of reference to carry on the implementation of growth and actualising steps… So far, conceptually, it would appear that my personal knowledge base, at this stage of my development, would constitute Psychodynamics, Dynamic Systems Theory and Positive Psychology.” In his explanation of how he looks at a client context, P5 notes: “As an assessment practitioner, the data tells you something and ‘it is what it is’.” Similarly, P7 rationalises his consulting skills in relation to a process model of consulting psychology; he seems to find comfort in the belief that the theoretical model will provide him with the ability to ensure success:

In the third stage, intervention phase, care should be given to ensure that the interventions to be implemented are a joint venture (joint action plan) between myself and the client and not necessarily my own prescriptions. The last stage, evaluation phase, is of importance for me because it allows me to be reflective and critical of myself and the consulting process in each stage, so as to address possible challenges and ensure that the consulting process brings about desired outcomes.

In these ways, students do not take ownership of their identity transformation, because of fearing their incompetence. They rather rationalise about how the programme will impart the knowledge they need, or how theoretical models will show them the way to be effective in consulting.

## 5. Discussion

This study aimed to develop an understanding of the coping dynamics that consulting psychology doctoral students employ when transitioning their professional role identity. In working with their professional identity, students show fear of incompetence as well as anxiety in relation to preserving the self. Such fear and anxiety are defined as performance and survival anxiety [30] and are typically also found in other system psychodynamic studies working with identity construction [26,45,72]. To deal with their performance and survival anxieties, the three themes constructed in the findings describe the students’ coping dynamics from a system psychodynamic stance in terms of basic assumption or anti-task behaviour, primary and sophisticated psychodynamic defences.

Students firstly engaged in basic assumption or anti-task behaviour such as dependency, fight, pairing and me-ness as a way of coping with their performance and survival anxieties. In doing so, they attempted to regain a sense of competence and self-efficacy while transitioning their professional role identity. In idealising consulting psychology, as well as the doctoral programme and its lecturers, students demonstrated a dependency mentality to resolve the insecurity and self-doubt they felt in having to take up the role of consulting psychologist. Similarly, pairing with these same objects, which they deemed powerful and authoritative, helped them to cope with feeling incompetent. Students therefore sought out the consulting psychology knowledge domain and the doctoral programme with its lecturers, as a container of their performance anxiety. To preserve the self and deal with survival anxieties in taking up the consulting psychology role, students reverted to an anti-task mentality of fight and me-ness. By attacking the task of self-reflection (i.e., the task of identity work) and resisting teamwork, students tried to preserve a sense of self that they knew and felt comfortable with. In doing so, they split the self from the task or from the team and projected onto the task/team the feelings of discomfort and suspicion related to their ability to be a consulting psychologist. By emphasising me-ness, students also attempted to preserve the self in relation to the identity tension they experienced between wanting to belong to the consulting psychology fraternity and retaining their unique psychologist identity.

Anxiety that resulted from perceived identity incongruences in their normative and existential or phenomenological role parts secondly surfaced in how they used projection, splitting and projective identification to deal with the ensuing fears of not being good enough. Taking up a new role entails taking up a role that is given or normative, which refers to the rational and measurable work related to the task [26,73]. At the same time, it includes taking up an informal role, reflected in the personal, frequently unconscious needs and aspirations of the individual [50]. The informal or unconscious role is also referred to as the existential or phenomenological role parts [26]. Students demonstrated these primary psychodynamic defences specifically in relation to coping with the incongruences they experienced in their formal or conscious task and the informal/unconscious task to manage the self in relation to the other. Through splitting the domains and roles of academic/scientist and practitioner, students coped with the tension of not feeling good enough in their existential/phenomenological role. Their self-doubt was recognised in the high expectations they introjected in relation to the consulting psychology role and the concurrent projective identification of not being good enough. Students continued to cope with their performance anxiety by projecting negative attributes such as narcissism and inefficiency onto others in an effort to preserve their sense of self.

Thirdly, they rationalised and intellectualised their work identity and professional role transition, in an attempt not to deal with the unwanted emotions that resulted from their identity work. Further expression of their normative role was also found in students’ intellectualisation and *rationalisation* of consulting psychology as an all-powerful knowledge domain. Through these two sophisticated defences, students talked about their consulting psychology role in a way that emphasised absolute knowledge and competence, ultimately demonstrating incongruence with their felt (introjected) sense of incompetence. In this way, reasoning and rational thought were used to avoid dealing with difficult emotions [37,74].

The findings show how consulting psychology doctoral students engage with and express identity work when they reflexively engage and formulate self-reflective thoughts about their experience of taking up a new professional role identity. It is hypothesised that active identity work (such as in self-reflective activities) is valuable for students to deal with their performance and survival anxiety, because in doing so, they uncover their unconscious coping dynamic, a dynamic that is a normal part of facilitating their adjustment to transitioning into a new professional identity. This finding is in support of reflective spaces or identity workspaces, which according to system psychodynamic theory provide a safe space to surface and consciously work with below-the-surface anxieties that result from identity transition [54,55]

Employing certain defence mechanisms to cope with performance anxiety has traditionally been described as maladaptive or pathological [35]. In the present study, the defensive coping dynamic that was evident in the students’ identity work is rather proposed to be a natural (normal) and evolving process of transitioning into a new professional role [54]. Some psychodynamic perspectives on coping emphasise hierarchies of defensive coping, depending on the level of reality distortion—from psychotic and immature to mature [70,75]. These theories show that coping *evolves* as a person’s cognitive functions mature [76]. This study further supports the notion of defences as behavioural phenomena, rather than referring to defence mechanisms as measurable constructs [77]. It is therefore conjectured that defensive coping is an important, dynamic and interrelated behavioural phenomenon that could potentially be conducive to adjustment, because it is a natural part of identity work. Defensive coping is an important part of understanding the coping phenomenon holistically, and it is in working with all the parts of the coping dynamic that the whole adjustment process can be facilitated. Defensive coping is conducive to adjustment, as it progresses the identity work relevant to professional role transition and brings to the surface the otherwise unconscious coping dynamic. Recently, even quantitative studies exploring the correlation between coping and defences have confirmed that specific adaptive strategies can only be effectively employed when unconscious processes have been attended to [78]. Active identity work is a way to develop consciousness of the self when taking up a new professional role.

Therefore, rather than speaking of either adaptive or immature coping strategies and either psychotic or mature defence mechanisms, coping is a dynamic phenomenon that includes defensive coping. Studies show that people constantly re-evaluate and reconstruct the self in the work context [72]. Unearthing the unconscious dynamics of defensive coping through conscious identity work may develop the resilience required to establish a professional identity in which the self is both interdependent and unique. A study of the systems psychodynamic role identity of academic supervisors [26] similarly demonstrates how focusing solely on conscious behavioural adaptation can limit valuable insight into unconscious adaptive identity work. We argue that the human coping phenomenon is studied only in part if the covert and unconscious social dimensions of coping, such as splitting, projections and projective identification, are ignored. The system psychodynamic stance, with its focus on the unconscious and irrational forces in human behaviour, moves beyond the mainstream cognitive coping theory that focusses on rational, conscious coping, by taking a depth perspective on this phenomenon. To focus solely on conscious behavioural adaptation may thus limit valuable insight into unconscious adaptive identity work.

The findings also have implications for the work of faculty in general and educators in particular. It is evident that students should be provided with more conscious and structured support. Firstly, it would serve to sensitise (creating awareness) faculty and educators to the anxiety-provoking realities of identity transition and identity formation. Secondly, it would create safe, contained spaces for conscious reflection nestled in the different components of the consulting psychology doctoral programme. Finally, identity work, in the form of self-reflective activities, should become the norm (rather than the exception), as a way of developing consciousness of the self. In doing this, coping would be appreciated as a dynamic phenomenon, and in the process, resilience would be nurtured as required in transitioning a new professional identity.

A system psychodynamic perspective to coping would be incomplete if the discussion does not allow for constructing interpretations and hypotheses of the individual as reflective of the larger system and the dilemmas and challenges faced by it [71]. This study therefore also has implications for understanding how the psychology profession in South Africa may be coping with its unresolved identity tensions as a collective. The students’ identity work reflects the performance and survival anxieties evident in the psychology profession, as their defensive coping mirrors similar dynamics in the profession as a collective. From the background to this article, the splitting of professional categories, fight behaviour, pairing with the legal system and intellectualisation and rationalisation through the formulation and reformulation of regulations demonstrate how the psychology profession is coping with its identity tensions and are suggestive of an identity transition. Like the students’ experiences, the psychology profession will have to find ways to “normalise” this tension (both a challenge and an opportunity) in search of an identity that is relevant and dynamic, given the ever-changing socio-political and economic landscape and changing societal needs.

This study is limited in the extent to which the range of the coping dynamic has been discussed, purely because of the limited scope of taking a specific approach. As such, the authors acknowledge that the findings reflect a specific stance that does not necessarily demonstrate the holistic coping dynamic we are advocating, because it does not deal with overt, cognitive coping strategies. As researchers we acknowledge the limitation associated with small sample sizes and qualitative analysis, and therefore, we do not claim that our findings constitute an absolute or generalisable truth. In true hermeneutic phenomenological fashion, we present the findings as a perspective that may add value to scholarly understanding in working with identity conflicts and related coping dynamics. The value of the psychodynamic approach to coping was celebrated in the findings and highlighted defensive coping as an essential and natural part of the whole coping dynamic. We support research proponents of in-depth, qualitative inquiry into the study of defensive coping [77]. Continuous interpretive inquiry from a psychodynamic stance, to build theory abductively [77], is recommended to enhance our understanding of defensive coping as part of adjustment rather than pure evidence of maladaptive coping. In this vein, we recommend research exploring students’ identity work as it evolves in their self-reflective work throughout the year, exploring the aspect of maturation in the phenomenon of defensive coping.

## 6. Conclusions

In general, consulting psychology doctoral students perform well in terms of coping with their professional identity development, as they have completed their first year of doctoral studies successfully and are all well on track towards completing the degree. An exploration of below-the-surface dynamics reveals an interesting defensive coping dynamic that contributes to a more holistic understanding of coping with identity transition in the psychology profession. Consciously, the students engaged in self-reflection about taking up the role of consulting psychologist in a rational and intelligent manner. Unconsciously, while transitioning a professional role identity, they experienced performance and survival anxiety, which became conscious in the process of self-reflection or identity work and in exploring their coping dynamics from a systems psychodynamic perspective. The students also mirrored the identity work and defensive coping of the larger psychology profession. The identity work of the individual professional cannot be detached from the identity work of the collective system but rather provides potential insight into the collective on its adaptive functioning.

System psychodynamic coping is a dynamic process phenomenon that should not be conceptually limited to the discussion of defence mechanisms. Coping with identity tensions and demands includes defensive coping, which is a natural phenomenon and something that is engaged with in everyday life as our professional careers develop. Conscious and active identity work reveals the related unconscious dynamics. The task of self-reflection not only propels the students to do identity work, but in the process, they become aware of their defensive coping and continue to develop reflexivity and resilience in transitioning to a new professional identity. Proponents of system psychodynamics view wellness as a relational and systemic concept [68]. A certain level of congruence between the internal and external reality of the self is needed for wellness to be developed and sustained. This wrestling with identity tension is therefore not only important, but necessary as well. In becoming aware of their identity-related anxieties and defensive coping, professionals, such as the students in this study, can feed this awareness back into the profession and collectively start to model the courage to acknowledge insecurities, take back their projections and repair splits. Psychological well-being and coping result from healthy intrapersonal (within a person) and interpersonal (between people) relations [54].

Developing consulting psychology competence is valuable in facilitating behavioural change in organisations [79,80]. The doctorate in consulting psychology is particularly important in the South African context to bridge professional divides in the psychology profession and draw from the multidisciplinary pool of skills and competence to continue to address the country’s mental health needs effectively on all levels.

## Figures and Tables

**Table 1:** Dependency behaviour as seen in the data cited.

Condensation	Verbatim Excerpts	Participant
Expectation that the programme and SP knowledge will bring about change, opportunities and success edge for her	My main objective with doing specifically this programme is to become more skilled … I can see the potential that it holds for the field I am working in, and it makes me very excited. I think using this approach can facilitate change that is required in this field and also provide me with an edge as consultant. I can’t help but to wonder how things will change? Will it be a marginal change, or will it open a new and exciting world with new opportunities for me?	P1
Relates PhD and CP to mother and expresses gratitude for being developed through the programme. Hero-ing the programme; finding it a safe space like “mother”	My assumptions were largely drawn from my Mother’s experience and interaction with her (she is a consulting psychologist) ... There is an obvious soft skill curriculum that comes with attaining the highest form of academic qualification, and in this case, I feel that it, my PhD, will supplement my development nicely.	P3
Dependency is demonstrated through anger at the challenging workload of the programme	I sometimes also feel that the PhD journey that I commenced this year, by registering in the programme, is demanding and stressful with regards to time and workload; however, it has always been my dream to pursue and complete my PhD. It feels as though I do not have enough time to see that my work as a student is always attended to timeously.	P2
Coping with performance anxiety by pairing with the supervisor, fantasy that the supervisor will enable academic success for her	Furthermore, having Professor XX as my research supervisor and the research module co-ordinator has enabled me to have more faith in my own capabilities and strengths.	P2
Finds solace in the power of the programme to help him cope with his limitations	I am thus grateful for this course because it has made me aware of this possible limitation and it thus affords me an opportunity to identify similar feelings of discomfort should they arise in future whilst I am engaged in consulting work—I would then come up with a strategy to either counsel myself to attend to those uncomfortable issues or perhaps ask a suitable colleague to assist me in that regard	P7
Relies on the consulting process to enable his success	However, because I am aware of this possible limitation, I will pay special attention to my interpretations during this stage and put in measures to minimise my biases in order to ensure that a more accurate picture of the client’s situation is upheld. Luckily, the consulting process itself (e.g., evaluation phase, stage 4) offers one the opportunity to evaluate their actions in each stage.	P7
Finds the programme supportive of his functioning	However, I must say that after a great exposure through this programme (training as a consulting psychologist) I have come to appreciate that as psychologist, we can help each other through the sharing or exchange of knowledge.	P4
Coping with her low self-confidence by looking to the programme to address her insecurities	I took it upon myself to apply for a consulting psychology programme for professional and academic development and as a challenge to myself to try and succeed in something outside of my scope of practice and the confines of clinical psychology. I have always looked down upon myself and with very low self-esteem.	P8
Idealising the programme as saving her from potential limited way of thinking (performance anxiety—what I know/don’t)	Consulting Psychology is different. It has allowed to me adopt a new and different frame of perspective and reference. I am now thinking in a broader and organisational environment.	P8

## References

[1] Laher S. Scope of Practice: Boon or Bane?. Psytalk..

[2] Pretorius G. (2012). Reflections on the scope of practice in the South African profession of psychology: A moral plea for relevance and a future vision. South Afr. J. Psychol..

[3] Van Zyl L.E., Nel E., Stander M.W., Rothmann S. (2016). Conceptualising the professional identity of industrial or organisational psychologists within the South African context. SA J. Ind. Psychol..

[4] Flax M. Are Definitions of Practice Hindering the Psychology Profession?. https://www.sacap.edu.za/blog/counselling/mental-health-care-in-south-africa/.

[5] HPCSA Update on the Review of the Regulations Relating to the Scope of the Profession of Psychology. https://www.psyssa.com/update-on-the-review-of-the-regulations-relating-to-the-scope-of-the-profession-of-psychology/.

[6] Department of Health (2019). Government Notice R1169: Notice not to Proceed with the Proposed Regulations Defining the Scope of the Profession of Psychology.

[7] Stets J.E., Serpe R.T., DeLamater J., Ward A. (2013). Identity Theory. Handbook of Social Psychology.

[8] Pratt M.G., Rockmann K.W., Kaufmann J.B. (2006). Constructing professional identity: The role of work and identity learning cycles in the customization of identity among medical residents. Acad. Manag. J..

[9] Breakwell G.M. (2015). Coping with Threatened Identities.

[10] Alvesson M. (2010). Self-doubters, strugglers, storytellers, surfers and others: Images of self-identities in organization studies. Hum. Relat..

[11] Brown A.D. (2014). Identities and identity work in organizations. Int. J. Manag. Rev..

[12] Kreiner G.E., Hollensbe E.C., Sheep M.L. (2006). Where is the “Me” among the “We”? Identity work and the search for optimal balance. Acad. Manag. J..

[13] Snow D.A., Anderson L. (1987). Identity work among the homeless: The verbal construction and avowal of personal identities. Am. J. Sociol..

[14] Sveningsson S., Alvesson M. (2003). Managing managerial identities: Organizational fragmentation, discourse and identity struggle. Hum. Relat..

[15] Pals J.L. (2006). Narrative identity processing of difficult life experiences: Pathways of personality development and positive self-transformation in adulthood. J. Pers..

[16] Ibarra H., Barbulescu R. (2010). Identity as narrative: Prevalence, effectiveness, and consequences of narrative identity work in macro work role transitions. Acad. Manag. Rev..

[17] Kirpal S. (2004). Researching work identities in a European context. Career Dev. Int..

[18] Saayman T., Crafford A. (2011). Negotiating work identity. SA J. Ind. Psychol..

[19] Brunning H. (2014). Psychoanalytic Essays on Power and Vulnerability.

[20] Stapley L. (2006). Individuals, Groups and Organizations Beneath the Surface: An Introduction.

[21] Bion W.R. (1961). Experiences in Groups.

[22] Cilliers F. (2018). The experienced impact of systems psychodynamic leadership coaching amongst professional in a financial service organisation. SA J. Eco. Manag. Sci..

[23] Long S., Long S. (2013). Socioanalytic methodology. Socioanalytic Methods: Discovering the Hidden in Organanisations and Social Systeims.

[24] Krantz J., Could L., Stapley L.F., Stein M. (2001). Dilemmas of organizational change: A systems psychodynamic perspective. The Systems Psychodynamics of Organizations: Integrating the Group Relations Approach, Psychoanalytic, and Open Systems Perspectives.

[25] Barabasz A. (2016). Psychodynamic perspective of organizational change. Management.

[26] Cilliers F. (2017). The systems psychodynamic role identity of academic research supervisors. S. Afr. J. High. Educ..

[27] Clarke S., Hahn H., Hoggett P. (2008). Object Relations and Social Relations: The Implications of the Relational Turn in Psychoanalysis.

[28] Blackman J.S. (2004). 101 Defences: How the Mind Shield Itself.

[29] De Board R. (2014). The Psychoanalysis of Organisations: A Psychoanalytic Approach to Behaviour in Groups and Organisations.

[30] Steyn M., Cilliers F. (2016). The systems psychodynamic experiences of organisational transformation amongst support staff. SA J. Ind. Psychol..

[31] Lipgar R.M., Pines M. (2003). Building on Bion: Branches: Contemporary Developments and Applications of Bion’s Contributions to Theory and Practice.

[32] Gabriel Y., Carr A. (2002). Organisations, management and psychoanalysis: An overview. J. Manag. Psych..

[33] Cramer P. (1998). Coping and defense mechanisms: What’s the difference?. J. Pers..

[34] Vaillant G.E. (2011). Involuntary coping mechanisms: A psychodynamic perspective. Dialogues Clin. Neurosci..

[35] Aldwin C.M., Brustrom J., Gottlieb B. (1997). Theories of coping with chronic stress. Coping with Chronic Stress.

[36] Petriglieri G., Stein M. (2012). The unwanted self: Projective identification in leader’s identity work. Org. Stud..

[37] Balls M. (2015). Rationalisation and Intellectualisation. Altern. Lab. Anim..

[38] Vansina L.S., Vansina-Cobbaert M. (2008). Psychodynamics for Consultants and Managers.

[39] Czander W.M. (1993). The Psychodynamics of Work and Organisations.

[40] Lazarus R.S. (1993). Coping theory and research: Past, present, and future. Psychosom. Med..

[41] Du Plessis M., Martins N. (2019). Developing a measurement instrument for coping with occupational stress in academia. SA J. Ind. Psychol..

[42] Barnard A., Clur L., Joubert Y. (2016). Returning to work: The cancer survivor’s transformational journey of adjustment and coping. Int. J. Qual. Stud. Health Well-Being.

[43] Fennell P.A. (2001). The Chronic Illness Workbook: Strategies and Solutions for Taking back Your Life.

[44] Petriglieri G. (2020). A Psychodynamic perspective on identity as fabrication. The Oxford Handbook of Identities in Organizations.

[45] Cilliers F., Harry N. (2012). The systems psychodynamic experiences of first-year master’s students in industrial and organisational psychology. SA J. Ind. Psychol..

[46] Bollas C. (2017). The Shadow of the Object: Psychoanalysis of the Unthought Known.

[47] French R., Vince R., French R., Vince R. (2002). Learning, managing, and organizing: The continuing contribution of group relations to management and organization. Group Relations Management and Organization.

[48] Vince R. (2018). Institutional illogics: The unconscious and institutional analysis. Organ. Stud..

[49] Long S. (2019). The unconscious won’t go away—Especially in organisations. Org. Soc. Dyn..

[50] Sievers B., Beumer U., Long S., Newton J., Sievers B. (2006). Organisational role analysis and consultation: The organisation as inner object. Coaching in Depth.

[51] Alvesson M., Willmott H. (2002). Identity regulation as organizational control: Producing the appropriate individual. J. Manag. Stud..

[52] Knapp H.D. (1989). Projective identification: Whose projection-whose identity?. Psychoanal. Psychol..

[53] Krantz J., Long S. (2013). Work culture analysis and reflective space. Socioanalytic Methods: Discovering the Hidden in Organanisations and Social Systems.

[54] Petriglieri G., Petriglieri J.L. (2010). Identity workspaces: The case of business schools. Acad. Manag. Learn. Educ..

[55] Petriglieri G., Petrilieri J.L., Wood J.D. (2018). Fast tracks and inner journeys: Crafting portable selves for contemporary careers. Admin. Sci. Q..

[56] Crowther S., Ironside P., Spence D., Smythe L. (2016). Crafting stories in hermeneutic phenomenology research: A methodological device. Qual. Health Res..

[57] Davidsen A.S. (2013). Phenomenological approaches in psychology and health sciences. Qual. Res. Psychol..

[58] Laverty S.M. (2003). Hermeneutic phenomenology and phenomenology: A comparison of historical and methodological considerations. Int. J. Qual. Methods.

[59] Tan H., Wilson A., Olver I. (2009). Ricoeur’s theory of interpretation: An instrument for data interpretation in hermeneutic phenomenology. Int. J. Qual. Methods.

[60] Kafle N.P. (2013). Hermeneutic phenomenological research method simplified. Bodhi Interdiscip. J..

[61] Cunliffe A.L. (2016). Republication of “On Becoming a Critically Reflexive Practitioner”. J. Manag. Educ..

[62] Giampapa F., Preece S. (2016). The politics of researcher identities: Opportunities and challenges in identities research. The Routledge Handbook of Language and Identity.

[63] Petriglieri G. (2020). F**k science!? An invitation to humanize organization theory. Organ. Theory.

[64] Nagata A.L. (2004). Promoting self-reflexivity in intercultural education. J. Intercult. Commun..

[65] Zietsma C., Toubiana M. (2018). The valuable, the constitutive, and the energetic: Exploring the impact and importance of studying emotions and institutions. Organ. Stud..

[66] Lindseth A., Norberg A. (2004). A phenomenological hermeneutical method for researching lived experience. Scand. J. Caring Sci..

[67] Churchill S.D. (2018). Explorations in teaching the phenomenological method: Challenging psychology students to “grasp at meaning” in human science research. Qual. Psychol..

[68] Henning S., Cilliers F. (2012). Constructing a systems psychodynamic wellness model. SA J. Ind. Psychol..

[69] Turquet P.M., Gibbard G.S., Hartman J.J., Mann R.D. (1974). Leadership—The individual in the group. Analysis of Groups.

[70] Vaillant L.M. (1997). Changing Character: Short-Term Anxiety-Regulating Psychotherapy for Restructuring Defences, Affects, and Attachment.

[71] Nagel C. (2020). Psychodynamic Coaching: Distinctive Features.

[72] Mayer C.-H., Tonelli L., Oosthuizen R.M., Surtee S. (2018). ‘You have to keep your head on your shoulders’: A systems psychodynamic perspective on women leaders. SA J. Ind. Psychol..

[73] Green Z.G., Molenkamp R.J. (2005). The BART System of Group and Organizational Analysis: Boundary, Authority, Role and Task. https://www.semanticscholar.org/paper/The-BART-System-of-Group-and-Organizational-Role-Green-Molenkamp/29cdfab7f00f8b4990e802bdad813a6ac750f018.

[74] Mayer C.-H., Flotman A.-P., Mahadevan J., Mayer C.-H. (2017). Constructing identity. Muslim Minorities, Workplace Diversity and Reflexive HRM.

[75] Haan N., Goldberger L., Breznitz S. (1993). The assessment of coping, defense, and stress. Handbook of Stress: Theoretical and Clinical Aspects.

[76] Radnitz C.L., Tiersky L., Martz E., Livneh H., Wright B. (2007). Psychodynamic and cognitive theories of coping. Coping with Chronic Illness and Disability.

[77] Mihalits D.S., Codenotti M. (2020). The conceptual tragedy in studying defense mechanisms. Integr. Psychol. Behav. Sci..

[78] Firoozi M. (2020). Interaction of conscious and unconscious mechanisms in coping with chronic pain. Anesthesiol. Pain..

[79] Falender C.A., Shafranske E.P., Falender C.A., Shafranske E.P. (2020). Consultation in psychology: A distinct professional practice. Consultation in Psychology: A Competency-Based Approach.

[80] Lowman R.L. (2016). An Introduction to Consulting Psychology: Working with Individuals, Groups, and Organizations.

